# Menopausal hormone therapy does not improve some domains of memory: A systematic review and meta-analysis

**DOI:** 10.3389/fendo.2022.894883

**Published:** 2022-09-06

**Authors:** Lin Chen, Wei Zheng, Gang Chen, Lin-Hua Liu, Jin Yao, Yan Chen

**Affiliations:** ^1^ Department of Internal Medicine, Fujian Provincial Hospital South Branch, Fuzhou, China; ^2^ Shengli Clinical Medical College, Fujian Medical University, Fuzhou, China; ^3^ Department of Neurology, Fujian Provincial Geriatric Hospital, Fuzhou, China; ^4^ Department of Endocrinology, Fujian Provincial Hospital, Fuzhou, China

**Keywords:** menopausal hormone therapy, memory, cognition, menopause, women, systematic review, meta-analysis

## Abstract

**Background:**

Aged women appear to be at a higher risk of developing memory impairment than men. Whether menopausal hormone therapy (MHT) could improve memory in postmenopausal women remains unclear. We thus conducted a meta-analysis to investigate the potential effect of MHT on memory, especially verbal memory, in postmenopausal women.

**Methods:**

PubMed, Cochrane, Embase, Chinese Biomedical Literature Database, and web of ClinicalTrials.gov were systematically searched for randomized controlled trials comparing MHT versus placebo in postmenopausal women. Our primary outcome of interest is memory function.

**Results:**

We included 10 studies with 2,818 participants in the final analysis. There was no significant differences in immediate recall (weighted mean difference [WMD] 0.34, 95% confidence interval [CI]: -0.73, 1.40), delayed recall (WMD 0.99, 95% CI: -0.51, 2.48), short-delay (WMD -0.00, 95% CI: -0.37, 0.37), and long-delay (WMD -0.19, 95% CI: -0.69, 0.31) recall between WMT and placebo. WMT was associated with a lower digit span forward (mean reduction -0.20, 95% CI: -0.36, -0.03). In women within 5 years of menopause, MHT did not differ in immediate (0.45, 95% CI: -0.75, 1.65) or delayed recall (1.03, 95% CI: -0.93, 3.00), and digit span forward (-0.11, 95% CI: -0.72, 0.50), when compared with placebo.

**Conclusion:**

This meta-analysis suggested that MHT had no effect on verbal memory in postmenopausal women, and may impair some domains of short-term memory. Current available evidence does not support MHT for improving memory in women less than 60 years, even in recently menopausal women.

**Systematic Review Registration:**

https://www.crd.york.ac.uk/PROSPERO, identifier CRD42021233255.

## Introduction

Alzheimer’s disease (AD) is the most common cause of dementia, occurring primarily in the elderly, and usually manifests initially as memory deficits. The incidence of AD has been reported to be higher in older women than in men (1), which may be related to declining estrogen levels (2, 3). Studies in animals and *in vitro* have shown that estrogen can protect brain structures, including those relevant to memory processes (4).

Some epidemiological studies suggest that menopausal hormone therapy (MHT) may be protective against cognitive decline and prevent dementia in postmenopausal women (5–7). However, the Nurses’ Health Study, which followed 13,807 women aged 70–81 years, found that long-term hormone users (at least 5–10 years) had an increased risk of cognitive decline in general cognition and verbal memory (8). The results of the effect of MHT on memory, especially on verbal memory, were inconsistent. In some studies (9, 10), women who received MHT appeared to have better performance on verbal memory, whereas some large trials (8, 11) showed that hormone therapy (HT) did not improve verbal memory. The WHI Study of Cognitive Aging (WHISCA) (12) even found that the combination of estrogen and progestin had a negative impact on verbal memory in postmenopausal women. Then, the “critical window” hypothesis was proposed in attempt to explain the discrepancies observed in the studies about estrogen and cognition in postmenopausal women. According to this hypothesis, the effect of MHT on cognition is related to the time initiation for treatment. HT can reduce cognitive decline if it is administered promptly in early menopause but show no effect or may even be harmful when treatment is started several years after menopause (13).

Cognition includes a variety of processes from memory to executive function, with memory impairment being the most common initial clinical manifestation and earliest predictor of AD, especially verbal memory impairment. In view of these inconsistent conclusions and the unproven “critical window” hypothesis (14), we therefore did a systematic review and meta-analysis of randomized controlled trials (RCTs) to explore the effect of MHT on memory, especially verbal memory, in postmenopausal women compared to placebo.

## Methods

### Search strategy and selection criteria

This meta-analysis was reported in accordance with the PRISMA Statement and was registered with PROSPERO (Registration number: CRD42021233255).

We selected relevant studies by searching PubMed, Embase, Cochrane, CBM, and ClinicalTrials.gov from inception to 18 June 2022 without language restriction. We used Medical Subject Headings (MeSH) terms: “hormone replacement therapy,” “cognition,” “menopause,” and truncations and synonyms of the following text: “estrogen,” “progestogen,” “memory,” and “postmenopausal.” The detailed search strategies are shown in **Supplementary File 1**.

### Study selection and data extraction

We included studies based on the following criteria : (1) RCTs ; (2) participants were healthy postmenopausal women who had been spontaneously menopausal for at least 12 months or had ovariectomy ; (3) interventions arm was HT, including estrogen, progestin, or combined estrogen–progestin therapy, with placebo as control, and the duration was at least 2 weeks ; (4) the outcomes contained memory-related tests. Exclusion criteria were as follows : (1) studies did not assess the effects of MHT on memory ; (2) subjects were perimenopausal women or postmenopausal women with any of the following conditions: depression, cognitive decline, or use of drugs that influence cognition ; (3) the interventions were topical vaginal hormone, estrogen receptor modulator, and estrogen in combination with compounds other than progestin derived; and (4) studies using subjective questionnaire to measure memory function.

Two independent investigators screened and reviewed the full text of any potentially relevant studies to determine whether they were included in the study. Disagreement was resolved through discussion with a third investigator.

Our primary outcomes of interest were as follows: immediate and delayed recall of logical memory assessed using the Wechsler Memory Scale–Revised (WMS-R) test, short- and long-delayed recall assessed using the California Verbal Learning Test (CVLT), and digit span which represents short-term memory retention.

These two investigators independently extracted study design, participant characteristics, intervention information (type, dosage, and duration of HT), and post-intervention cognition outcome scores [mean (SD)]. We defined that the data at the first time point were selected for analysis, when the results of the study presented several periods of follow-up. If the post-intervention outcome of SDs was unavailable, we imputed them by borrowing the baseline SDs, assuming that the intervention does not alter the variability of the outcome measure (15). Trial quality was assessed by two independent reviewers using the Cochrane risk of bias tool (16).

### Statistical analysis

Random-effects models were used to calculate the pool estimated effect size (weighted mean difference [WMD] with 95% confidence intervals [CIs] for continuous variables. We performed prespecified subgroup analyses stratified by the length of menopause (>5 years vs. ≤5 years) and formulation of HT (estrogen-alone versus estrogen-plus progestogen). Heterogeneity of treatment effects between studies was assessed using the *I²* statistic, where *I²* values > 50% were considered substantial heterogeneity (17). We assessed the possibility of publication bias by constructing a funnel plot. Then, Egger tests were used to assess funnel plot asymmetry, with *P* < 0.1 considered as significant publication bias. For studies with missing data or substantial variability, we conducted sensitivity analyses with individual study removed from the pooled analysis to ensure the stability of results. All statistical analyses were performed with Revman 5.4 and Stata 16.0 software.

## Results

### Study characteristics

**Figure 1:**
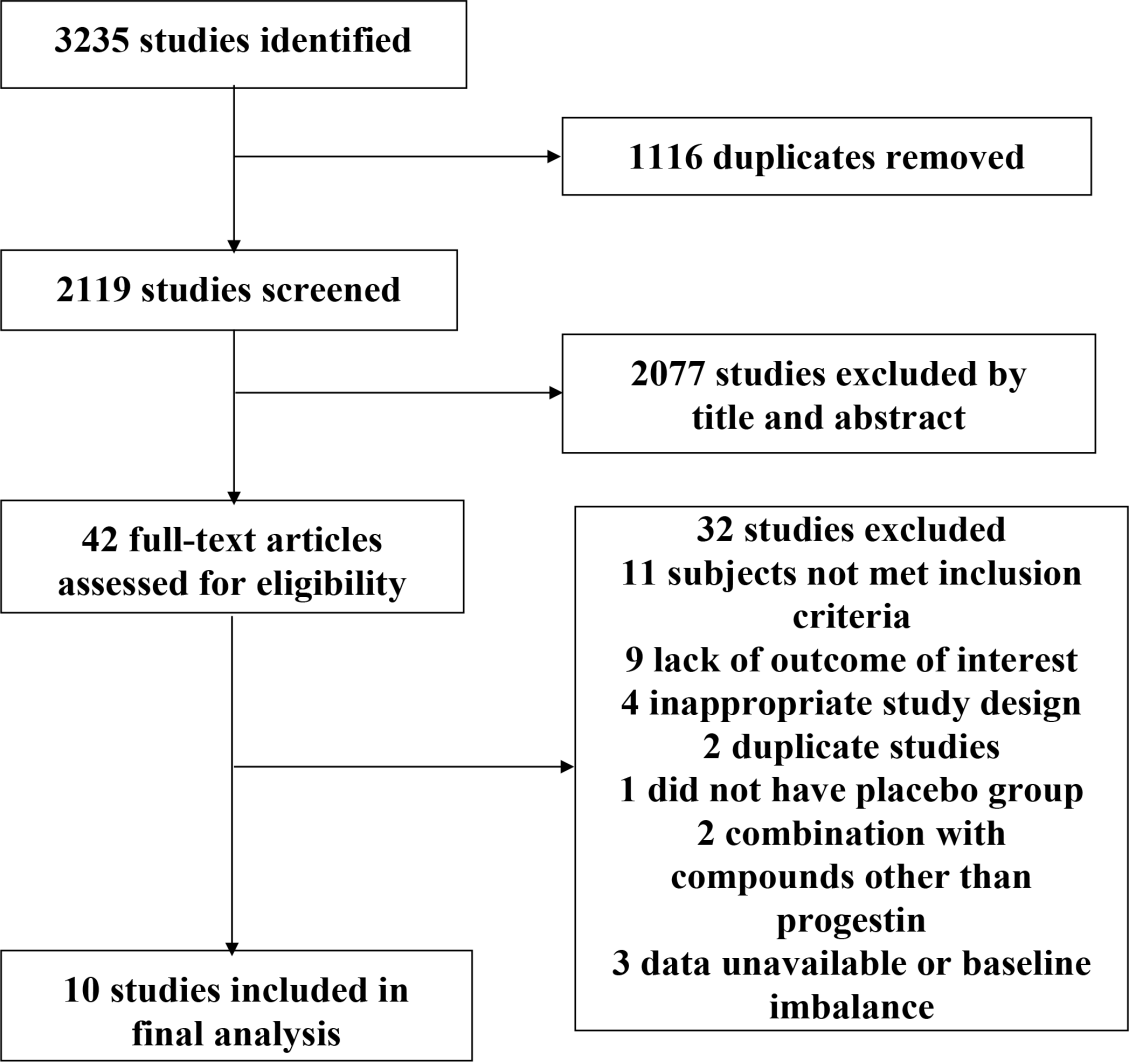
Flow diagram of study selection process.

We identified a total of 3,235 studies, of which 42 RCTs were potential eligible for inclusion. Ten studies enrolling 2,818 individuals were included in the final analysis (**Figure 1**). In one of the included studies (18), we used estimated SDs for pooled analysis, due to a lack of available SDs, while the other trials had accessible SD.

The 10 studies were all parallel groups designed and were published between 1992 and 2011, with the majority published after 2005 (**Table 1**). Three trials (18, 19, 26) included young postmenopausal women with mean ages of 48.2, 52.2, and 50.0, respectively, all with time since menopause less than 5 years. Seven studies (12, 20–25) included women with last menstrual cycle for more than 5 years. As for menopause manner, three studies (19, 24, 26) included surgically menopausal patients. Interventions in the trials included estradiol alone (19, 20, 22, 23, 26), estrogen–progestin combinations (12, 18, 24, 25), and a mixture of the above two treatments (21). Four studies (12, 18, 23, 26) prescribed oral-conjugated equine estrogen (CEE). Two studies (19, 25) used estradiol valerate treatment, either intramuscularly or orally. The other four studies (20–22, 24) used transdermal or oral 17-β estradiol. The mean trial duration was 48.1 weeks (range 10 weeks to 3 years). Six studies (18–22, 26) evaluated the effects of MHT on logical memory, and five studies (12, 18, 20, 23, 24) explored the outcomes of short-delay or long-delay recall. As for digit span, five trials (12, 18, 19, 23, 26) assessed digit span forward, four of which also assessed digit span backward, whereas the other three (20, 22, 25) assessed digit span total.

**Table 1:** Characteristics of included studies.

First author	Year	Type of menopause	N	Mean age	Treatment	Duration of intervention	Outcomes assessed	Mean time since menopause	Design
Phillips et al. (19)	1992	surgical menopause	19	48.2 years	monthly IM injections of 10 mg E_2_ valerate or placebo	3 months	immediate and delayed recall of logical memory, digit span	Immediately after bilateral oophorectomy	parallel-groups
Dunkin et al. (20)	2005	natural or surgical menopause	17	57 years	transdermal 17β-E_2_ released at 0.1 mg E_2_/d or placebo	10 weeks	immediate and delayed recall of logical memory, digit span	7.9 years	parallel-groups
Resnick et al. (12)	2006	natural menopause	1416	Over 65 years	Oral CEE (0.625 mg) with MPA(2.5mg) or placebo	3 years	short and long delayed recall from CVLT, digit span	At least 5 years	parallel-groups
Maki et al. (18)	2007	natural menopause	158	52.2 years	Oral CEE (0.625 mg) with MPA(2.5mg) or placebo	4 months	immediate and delayed recall of logical memory, short and long delayed recall from CVLT, digit span	21.1months	parallel-groups
Pefanco et al. (21)	2007	natural menopause	55	Over 65 years	Oral micronized 17β-E_2_ (0.25 mg/d)/with micronized progesterone(100 mg/d) or placebo	3 months	immediate and delayed recall of logical memory	At least 5 years	parallel-groups
Marinho et al. (22)	2008	natural menopause	65	54.1 years	Oral 17β-E_2_(2 mg) or placebo	12 weeks	immediate and delayed recall of logical memory, digit span	At least 5 years	parallel-groups
Resnick et al. (23)	2009	natural menopause	886	Over 65 years	Oral CEE (0.625 mg) or placebo	3 years	short and long delayed recall from CVLT, digit span	At least 5 years	parallel-groups
Tierney et al. (24)	2009	natural or surgical menopause	133	Over 60 years	Oral 17β-E_2_(1 mg) and norethindrone(0.35 mg) 3 days/week or placebo	1 years	short delayed recall from CVLT	At least 5 years	parallel-groups
Alhola et al. (25)	2010	natural menopause	16	62.9 years	Oral estradiol valerate (2mg) with norethisterone (0.7 mg)	6 months	digit span	12 years	parallel-groups
Gorenstein et al. (26)	2011	natural or surgical menopause	53	50 years	Oral CEE (0.625 mg) or placebo	24 weeks	immediate and delayed recall of logical memory, digit span	3.6 years	parallel-groups

IM, intramuscular; E_2_, estradiol; 17β-E_2_, 17β- estradiol; CEE, conjugated equine estrogen; MPA, medroxyprogesterone acetate; CVLT, California Verbal Learning Test.

### Study quality assessment

The assessment of risk of bias using the Cochrane risk of bias tool was shown in **Supplementary Image 1**. Randomization method and blinding of participants and personnel were conducted adequately in most studies. However, four studies (18–21) did not specify whether allocation concealment was performed, and four studies (19, 20, 22, 26) did not adequately report the blinding of outcome assessments. Three studies were classified as being at high risk for other bias, including two studies (12, 23) that terminated the intervention early due to the publication of findings from WHI, and another study (20) that lost the second plasma estradiol data due to changes in laboratory protocol.

### Pooled analyses

**Figure 2:**
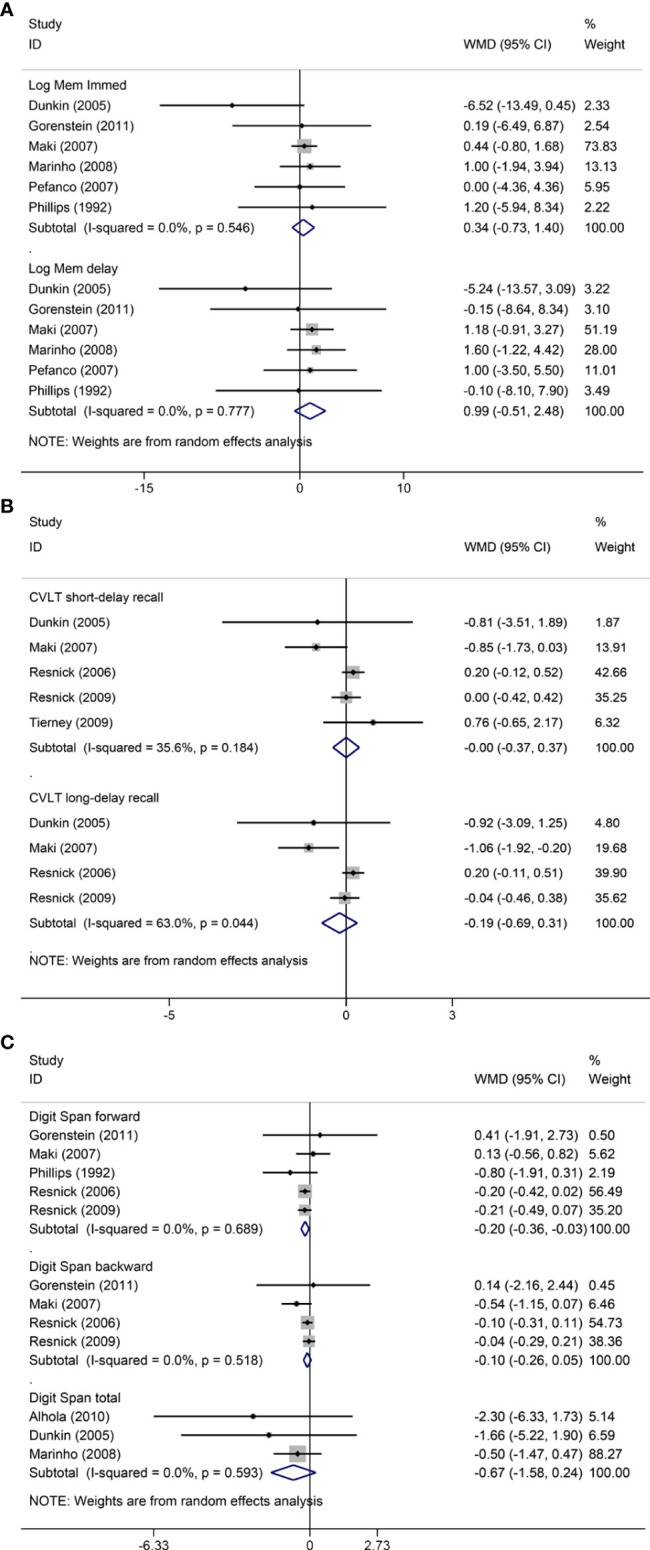
Meta-analyses of MHT versus placebo on comparing memory. Outcomes assessed are as follows: immediate and delayed recall of logical memory **(A)**, short- and long-delayed recall from CVLT **(B)**, and digit span including forward, backward, and total scores **(C)**. Log Mem Immed, immediate recall of logical memory; Log Mem delay, delayed recall of logical memory.

**Figure 3:**
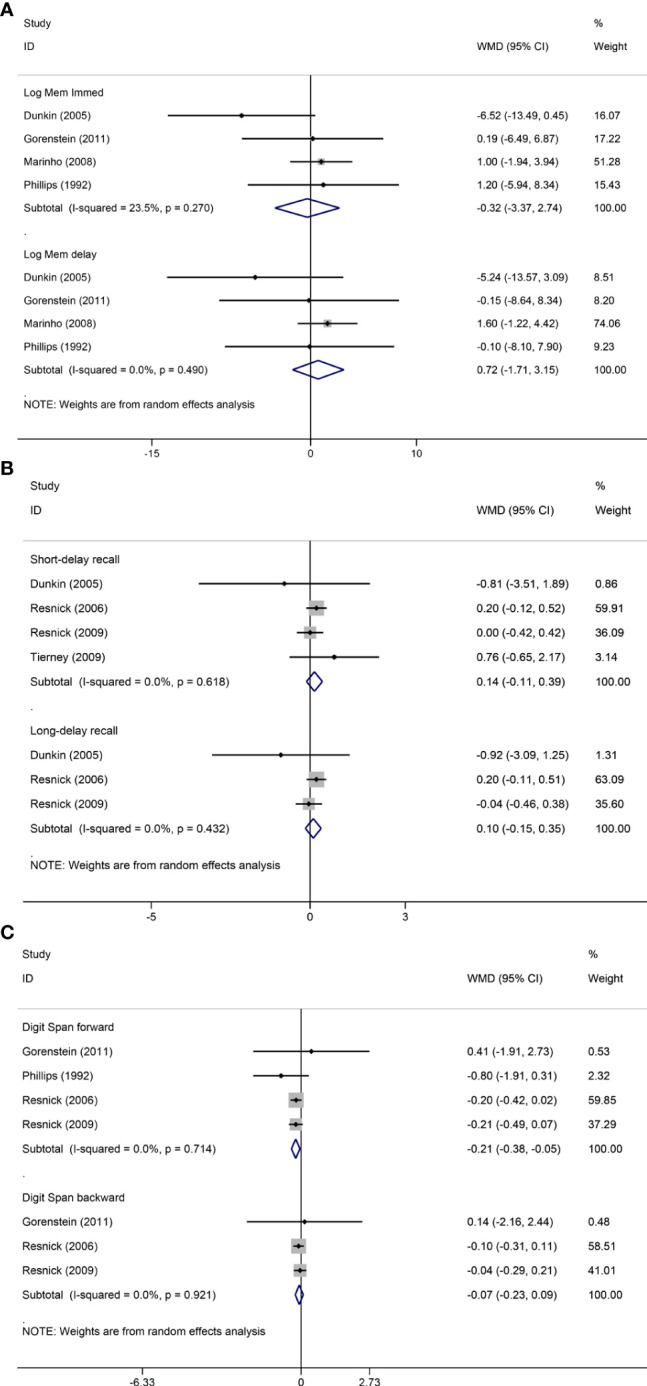
Sensitivity analyses of MHT versus placebo on comparing memory. Outcomes assessed are as follows: immediate and delayed recall of logical memory **(A)**, short- and long-delayed recall from CVLT **(B)**, and digit span including forward and backward **(C)**.

**Figure 4:**
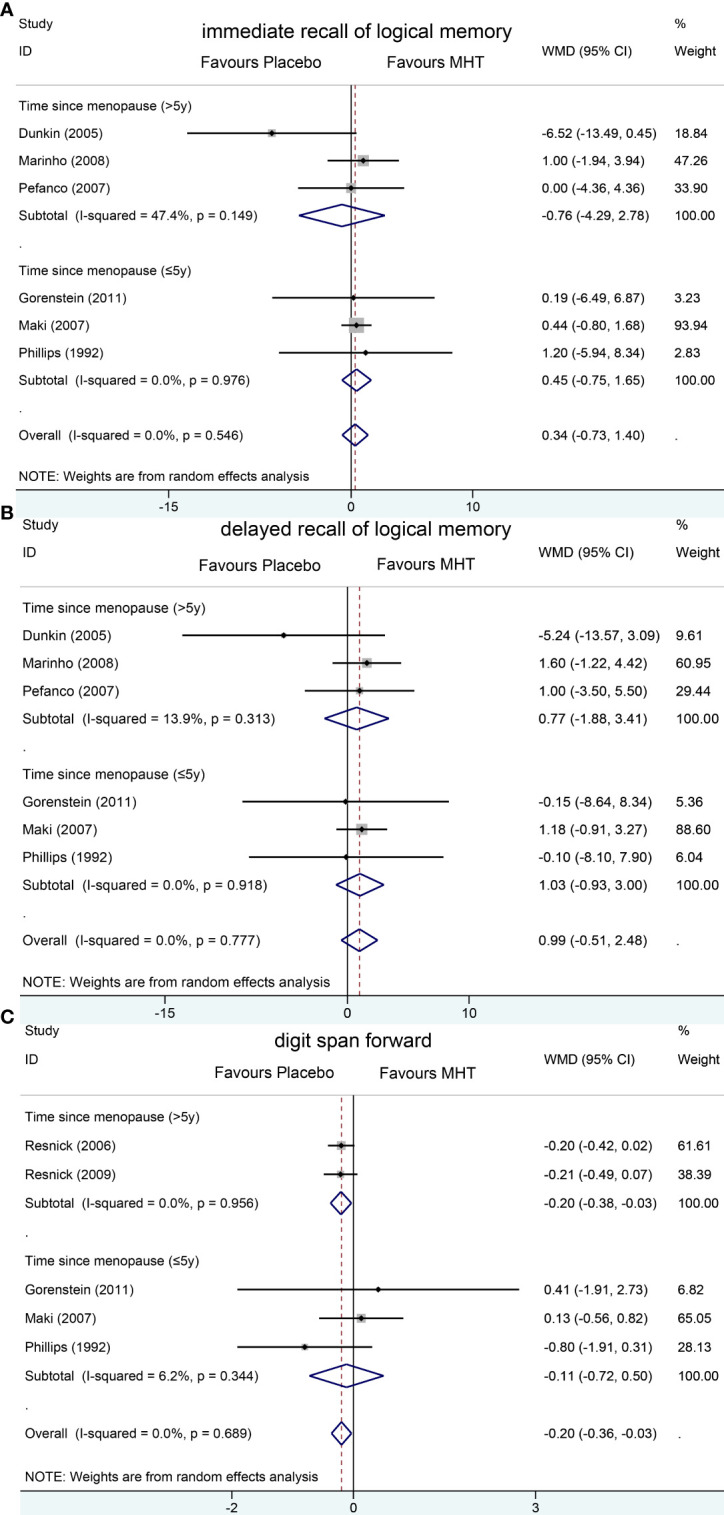
Subgroup analyses of MHT versus placebo on comparing memory according to the length of menopause. Outcomes assessed are as follows: immediate recall of logical memory **(A)**, delayed recall of logical memory **(B)**, and digit span forward **(C)**.

**Figure 5:**
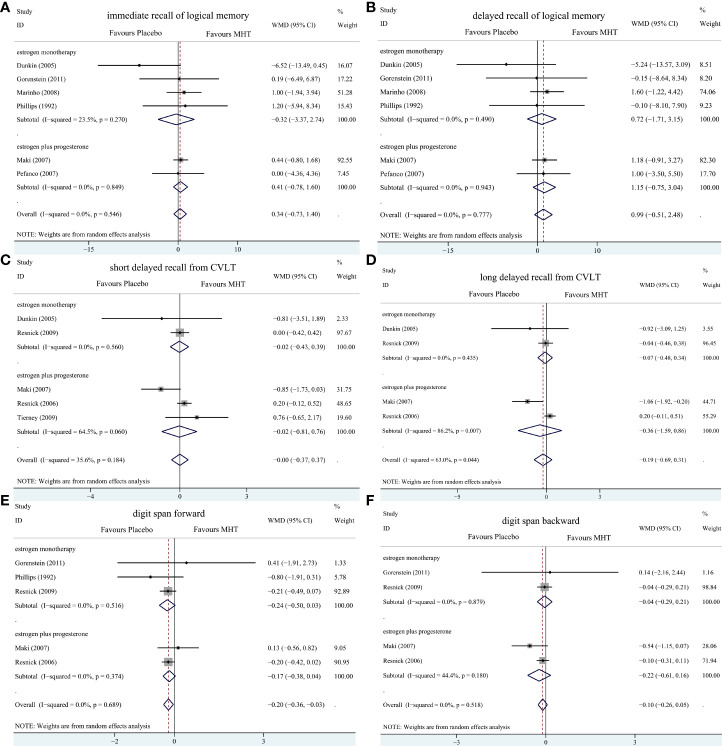
Subgroup analyses of MHT versus placebo on comparing memory stratified by the formulation of HT. Outcomes assessed are as follows: immediate recall of logical memory **(A)**, delayed recall of logical memory **(B)**, short delayed recall from CVLT **(C)**, long-delayed recall from CVLT **(D)**, digit span forward **(E)**, and digit span backward **(F)**.

Pooled analyses of six trials (18–22, 26) detected no significant differences in immediate and delayed recall of logical memory assessed using the WMS-R test between MHT and placebo (**Figure 2**). The funnel plot did not reveal significant asymmetry, and the Egger test showed no publication bias (*P* = 0.41; *P* = 0.12, respectively, **Supplementary File 2**). Two studies (18, 21) used estimated SD values or estimated sample size (two women dropped out before the 3-month evaluation, with an estimated loss of one in each group). Sensitivity analyses excluding these two studies did not alter our results (**Figure 3**). Further subgroup analyses showed that MHT and placebo did not differ in the immediate recall of logical memory for either women within 5 years of menopause (WMD 0.45, 95% CI: -0.75, 1.65) or more than 5 years (WMD -0.76, 95% CI: -4.29, 2.78, **Figure 4**). In the subgroup analyses stratified by formulations of hormones, both estrogen monotherapy (WMD -0.32, 95% CI: -3.37, 2.74, **Figure 5**) and estrogen–progestin combinations (WMD 0.41, 95% CI: -0.78, 1.60, **Figure 5**) had no beneficial or harmful effect on immediate recall of logical memory. MHT and placebo did not differ in the delayed recall of logical memory when stratified by early (≤5 years) or late (>5 years) stage of menopause (WMD 1.03, 95% CI: -0.93, 3.00; 0.77, 95% CI: -1.88, 3.41 respectively, **Figure 4**). Neither estrogen-alone (WMD 0.72, 95% CI: -1.71, 3.15, **Figure 5**) nor estrogen-plus progesterone (WMD 1.15, 95% CI: -0.75, 3.04, **Figure 5**) significantly improved the delayed recall. Pooled analyses of five (or four) studies that assessed the delayed recall from CVLT showed that MHT and placebo did not differ in the short- and long-delay recall (**Figure 2**). No significant funnel plot asymmetry and publication bias were detected (*P* = 0.54; *P* = 0.19, respectively, **Supplementary File 2**). Excluding the tudy that may cause high heterogeneity (18) yielded similar results regarding long-delay recall (WMD 0.10, 95% CI: -0.15, 0.35, *I^2^* = 0%, *P* = 0.43 **Figure 3**). The subgroup analyses stratified by the formulations of HT showed that either estrogen monotherapy or estrogen-plus progestin versus placebo did not differ in the short-delay and long-delay recall (**Figure 5**).

The participants who were treated with MHT had a reduction in digit span forward compared with placebo (WMD -0.20, 95% CI: -0.36, -0.03 **Figure 2**). No publication bias was detected regarding digit span forward and backward (**Supplementary File 2**). Publication bias for digit span total were not assessed because of the small number of studies (*n* = 3). Further sensitivity analysis by excluding study (18) using estimated SD values did not change our findings (**Figure 3**). Subgroup analysis showed that HT and placebo did not differ in digit span forward in women with early menopause (-0.11, 95% CI: -0.72, 0.50, **Figure 4**). For those with late menopause group, HT was associated with a reduction of digit span forward (-0.20, 95% CI: -0.38, -0.03, *I^2^* = 0.0%; **Figure 4**). Subgroup analysis stratified by the formulations of HT showed that neither estrogen-alone nor estrogen-plus progestogen treatment influenced digit span forward and backward compared with placebo (**Figure 5**).

## Discussion

The current meta-analysis showed that HT and placebo did not differ in the logical memory in postmenopausal women, even in recently menopausal women. In addition, MHT did not show beneficial or harmful effects on delayed recall of CVLT, digit span backward and total, but may have negative effect on digit span forward. These results suggest that MHT had no effect on verbal memory but may even decrease short-term memory retention.

Data from observational studies (6, 27–29) and randomized controlled studies (9, 30) suggested that postmenopausal women who took HT had better performance in verbal memory and a lower risk of AD. However, findings of the studies mentioned above were compromised due to the methodology flaw. For instance, some women had a recall bias about whether they were treated with estrogen. Moreover, women who used estrogen may be more concerned about their health and have a relatively healthier lifestyle, with a more pronounced effect on cognitive outcomes.

The results of RCT exploring the effects of MHT on cognitive change were inconsistent, including memory domain (9, 23). In our analyses, we focused on memory impairment, which has been shown to be the most common initial symptom and earliest predictor for AD. The WMS-R test is sensitive to the early diagnosis of AD. In addition, Albert (31) noted that delayed recall trial from CVLT was one of the significant predictors of cognitive impairment in AD. We finally referred to the outcome measures used in the Cochrane review (32) and selected the most frequently occurring and consistently administered tests from the included RCT as outcome measures.

A previous review (33) showed the beneficial effects of estrogen alone on memory in recently menopausal women. Sherwin (13) also found that the vast majority of the reviewed studies support the idea that early but not late initiation of MHT might prevent cognitive decline. However, subsequent large clinical trials have not found cognitive benefits from estrogen therapy (34) and even indicated that combined estrogen–progestin therapy decreased verbal memory in young postmenopausal women (18). In our study, the subgroup analyses stratified by time since menopause suggested no effect of HT on logical memory in recently menopausal women and old woman (menopause > 5 years). Although HT showed an overall deleterious effect on digit span forward in postmenopausal women, we found that, in women with early menopause, HT did not show harmful effects. In summary, our analyses suggested that HT may not affect the verbal memory or short-term memory retention of women in early menopause, which did not seem to support the “critical period” hypothesis (13).

The WHISCA and the Cognitive Complaints in Early Menopause Trial (COGENT) showed that CEE plus medroxyprogesterone acetate (MPA) worsened verbal memory (12, 18), whereas CEE alone did not (23). Some randomized trials revealed that estrogen alone (35–37) had no significant effect on cognition in postmenopausal women. These results indicated that estrogen versus estrogen–progestin may have different effects. However, the inclusion criteria for interventions in our study contained estrogen, progestin or combined estrogen–progestin therapy, considering the small amounts of studies were qualified. So, subgroup analyses were conducted, which showed that neither estrogen-alone nor estrogen-plus progestin treatment influenced verbal memory. The results were not consistent with the above studies. A possible reason might be the progestins in our included study were not only MPA but also micronized progesterone and norethisterone. A review which suggested that MPA had negative effect on cognition but other progestins might have neutral effects (38). However, the number of our included studies was insufficient to allow a further subgroup analysis stratified by different progestin or estrogen formulations. The potential effects of progestin on cognition need further research.

Moreover, our study has yielded findings that MHT provides no benefit on delayed recall from CVLT over placebo. In the analysis of long-delay recall, significant heterogeneity was considered to come from the trial of Maki (18). A possible explanation might be that the participants included in the study were young postmenopausal women (aged 45–55 years), which differs from other studies that included women over 65 years. The same conclusions can still be drawn with no heterogeneity between studies by excluding this study, which support the view that MHT does not impair verbal memory in older women.

With the publication of the WHI research results, most professional societies recommended against initiating MHT after age 60 years because of the higher risk of vascular events in this group. In our study, there was also no evidence that MHT was beneficial for memory in postmenopausal women in both early stage (≤5 years) and late stage (>5 years). Considering the risk of MHT suggested by the WHI research, the prescription of MHT to postmenopausal women less than 60 years for improving verbal memory and short-term memory retention should be cautious.

A limitation of this analysis is that studies included were few due to various cognitive tests used. The sample size in early menopause group is small, which may lead to the low power to detect a significant effect. It was also unable to further analyze whether factors such as the route of HT administration, estrogen or progestin types and dose, the manner of menopause, and the duration of intervention affect the results of the analysis. Some studies were short-term HT use. Second, some studies have potential risks of bias, including unclear allocation concealment and blind implementation, and even some trials have high risks of bias, including early termination of studies and incomplete data.

## Conclusions

This review suggested that HT had no impact on verbal memory in postmenopausal women, and may impair some domains of short-term memory. Current available evidence does not support that MHT should be prescribed for improving memory cognitive benefits in women less than 60 years, even in recently menopausal women. However, more high-quality large RCTs are needed in the future to further validate the effects of MHT on memory. It is hoped that future research focus on the population who are recently menopausal, head-to-head trials of different types of progestins and estrogens, and exploring the effects of MHT treatment duration.

## Data availability statement

The raw data supporting the conclusions of this article will be made available by the authors, without undue reservation.

## Author contributions

LC and WZ collected the data and performed the statistical analysis. LC and WZ were major contributors in writing the manuscript. GC designed the study and interpreted the data. L-HL was responsible for study supervision and coordination. JY and YC checked and revised the manuscript. All authors read and approved the final manuscript.

## Funding

This work was supported by grants from Startup Fund for scientific research, Fujian Medical University of China (Grant number:2017XQ1153).

## Acknowledgments

We thank all patients and their families involved in the study.

## Conflict of interest

The authors declare that the research was conducted in the absence of any commercial or financial relationships that could be construed as a potential conflict of interest.

## Publisher’s note

All claims expressed in this article are solely those of the authors and do not necessarily represent those of their affiliated organizations, or those of the publisher, the editors and the reviewers. Any product that may be evaluated in this article, or claim that may be made by its manufacturer, is not guaranteed or endorsed by the publisher.

## Supplementary material

Supplementary Image 1 Risk of bias summary.

## Abbreviations

MHT, Menopausal hormone therapy; AD, Alzheimer’s disease; WHI, Women’s health initiative; HT, Hormone therapy; RCT, Randomized controlled trial; WMS-R, Wechsler memory scale-revised; CVLT, California verbal learning test.
