# Supplementary material for: Menopausal hormone therapy does not improve some domains of memory: A systematic review and meta-analysis
Source: Front Endocrinol (Lausanne). 2022 Sep 6;13:894883. doi: 10.3389/fendo.2022.894883 (PMC9486389; doi:10.3389/fendo.2022.894883)
Supplement: Supplementary file 1 [file DataSheet_1.docx]

**The detailed search strategies used on pubmed:**

The complete search used for PubMed was: (((("hormone replacement therapy"[Mesh]) OR ((((((((therap*, hormone replacement[Title/Abstract]) OR (hormone replacement therapies[Title/Abstract])) OR (replacement therap*, hormone[Title/Abstract])) OR (estrogen*[Title/Abstract])) OR (oestrogen*[Title/Abstract])) OR (estradiol*[Title/Abstract])) OR (progesteron*[Title/Abstract])) OR (progestagen*[Title/Abstract]))) AND (("cognition"[Mesh]) OR (((((cognit*[Title/Abstract]) OR (quality of life[Title/Abstract])) OR (memor*[Title/Abstract])) OR (mood*[Title/Abstract])) OR (dementia[Title/Abstract])))) AND (("menopause"[Mesh]) OR ((postmenopaus*[Title/Abstract]) OR (post-menopaus*[Title/Abstract])))) AND (randomized controlled trial[Publication Type] OR randomized[Title/Abstract] OR placebo[Title/Abstract])
