# Supplementary material for: Menopausal hormone therapy does not improve some domains of memory: A systematic review and meta-analysis
Source: Front Endocrinol (Lausanne). 2022 Sep 6;13:894883. doi: 10.3389/fendo.2022.894883 (PMC9486389; doi:10.3389/fendo.2022.894883)
Supplement: Supplementary file 2 [file DataSheet_2.docx]

Table S1. Egger's test about studies of MHT on immediate recall from WMS-R

| Std_Eff Coef. Std. Err. t P>\|t\| [95% Conf. Interval] |
| --- |
| slope .9187609 .8000063 1.15 0.315 -1.302413 3.139934  bias -.5576232 .6016781 -0.93 0.406 -2.228149 1.112903 |

Table S2. Egger's test about studies of MHT on delayed recall from WMS-R

| Std_Eff Coef. Std. Err. t P>\|t\| [95% Conf. Interval] |
| --- |
| slope 2.456727 .8474004 2.90 0.044 .1039666 4.809488  bias -.9110281 .4538122 -2.01 0.115 -2.171013 .3489565 |

Table S3. Egger's test about studies of MHT on short-delay recall from CVLT

| Std_Eff Coef. Std. Err. t P>\|t\| [95% Conf. Interval] |
| --- |
| slope .2338254 .293348 0.80 0.484 -.6997389 1.16739  bias -.7473837 1.068729 -0.70 0.535 -4.148557 2.653789 |

Table S4. Egger's test about studies of MHT on long-delay recall from CVLT

| Std_Eff Coef. Std. Err. t P>\|t\| [95% Conf. Interval] |
| --- |
| slope .4861703 .2847299 1.71 0.230 -.7389236 1.711264  bias -2.264579 1.169408 -1.94 0.192 -7.296134 2.766976 |

Table S5. Egger's test about studies of MHT on digit span forward

| Std_Eff Coef. Std. Err. t P>\|t\| [95% Conf. Interval] |
| --- |
| slope -.2133291 .1220703 -1.75 0.179 -.6018112 .1751529  bias .120858 .6505533 0.19 0.864 -1.949493 2.191209 |

Table S6. Egger's test about studies of MHT on digit span backward

| Std_Eff Coef. Std. Err. t P>\|t\| [95% Conf. Interval] |
| --- |
| slope -.0310475 .1421759 -0.22 0.847 -.6427809 .5806858  bias -.5528597 .9013834 -0.61 0.602 -4.431199 3.32548 |
