# Supplementary figures and images for: Menopausal hormone therapy does not improve some domains of memory: A systematic review and meta-analysis
Source: Front Endocrinol (Lausanne). 2022 Sep 6;13:894883. doi: 10.3389/fendo.2022.894883 (PMC9486389; doi:10.3389/fendo.2022.894883)

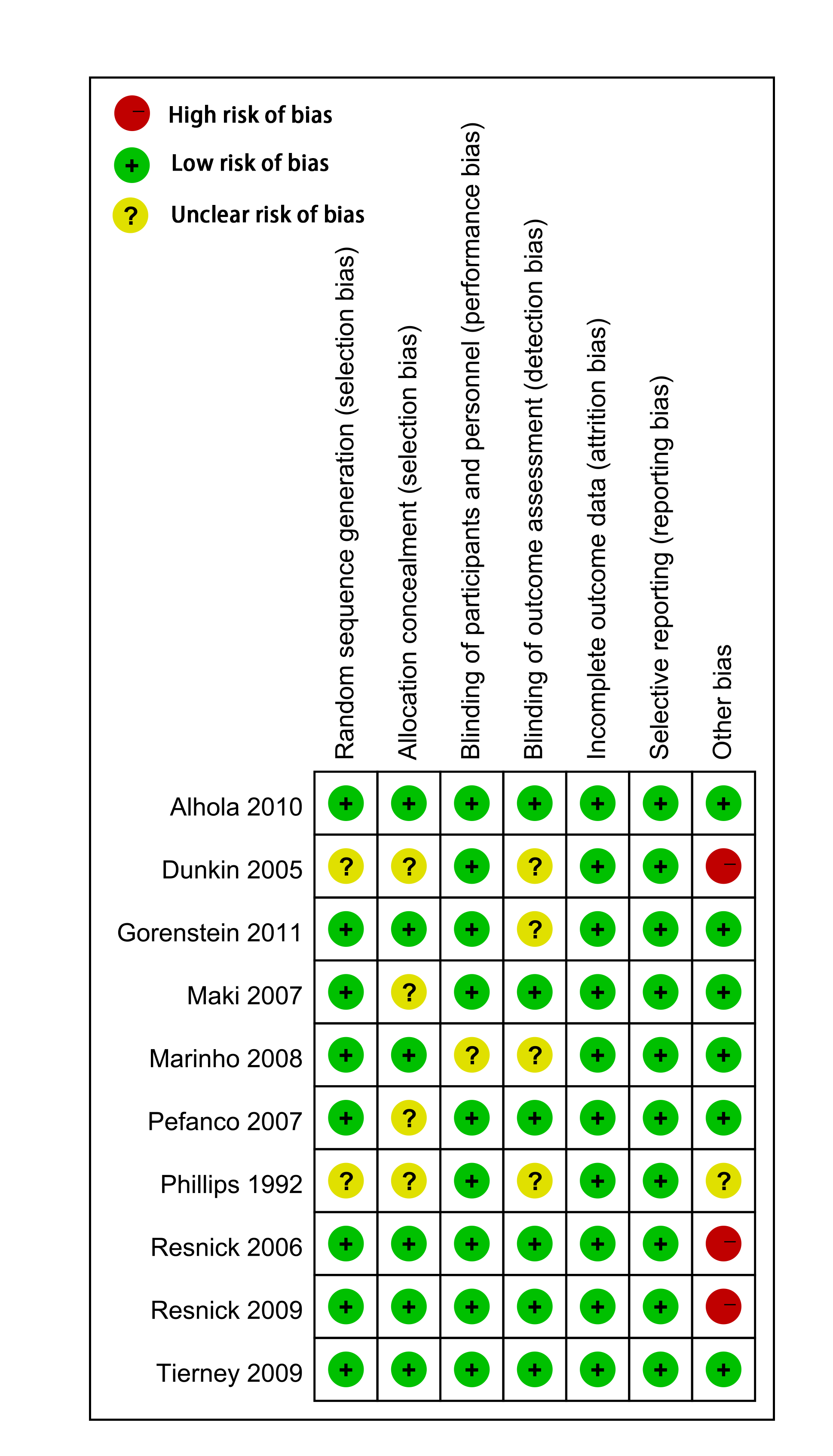

Supplement: Supplementary Image 1 — Risk of bias summary. [file Image_1.tif]
